# Olive Pomace-Derived Biomasses Fractionation through a Two-Step Extraction Based on the Use of Ultrasounds: Chemical Characteristics

**DOI:** 10.3390/foods10010111

**Published:** 2021-01-07

**Authors:** María del Mar Contreras, Irene Gómez-Cruz, Inmaculada Romero, Eulogio Castro

**Affiliations:** 1 Campus Las Lagunillas, Department of Chemical, Environmental and Materials Engineering, University of Jaén, 23071 Jaén, Spain; igcruz@ujaen.es (I.G.-C.); iromero@ujaen.es (I.R.); ecastro@ujaen.es (E.C.); 2 Center for Advanced Studies in Earth Sciences, Energy and Environment (CEACTEMA), University of Jaén, Campus Las Lagunillas, 23071 Jaén, Spain

**Keywords:** antioxidants, biorefinery, olive-derived biomass, ultrasound-assisted extraction, valorization

## Abstract

Olive-derived biomass is not only a renewable bioenergy resource but also it can be a source of bioproducts, including antioxidants. In this study, the antioxidant composition of extracted olive pomace (EOP) and a new byproduct, the residual fraction from olive pit cleaning (RFOPC or residual pulp) was characterized and compared to olive leafy biomass, which have been extensively studied as a source of antioxidants and other bioactive compounds with pharmacological properties. The chemical characterization showed that these byproducts contain a high amount of extractives; in the case of EOP, it was even higher (52.9%) than in olive leaves (OL) and olive mill leaves (OML) (35.8–45.1%). Then, ultrasound-assisted extraction (UAE) was applied to recover antioxidants from the extractive fraction of these biomasses. The solubilization of antioxidants was much higher for EOP, correlating well with the extractives content and the total extraction yield. Accordingly, this also affected the phenolic richness of the extracts and the differences between all biomasses were diminished. In any case, the phenolic profile and the hydroxytyrosol cluster were different. While OL, OML, and EOP contained mainly hydroxytyrosol derivatives and flavones, RFOPC presented novel trilignols. Other compounds were also characterized, including secoiridoids, hydroxylated fatty acids, triterpenoids, among others, depending on the bioresource. Moreover, after the UAE extraction step, alkaline extraction was applied recovering a liquid and a solid fraction. While the solid fraction could of interest for further valorization as a biofuel, the liquid fraction contained proteins, sugars, and soluble lignin, which conferred antioxidant properties to these extracts, and whose content depended on the biomass and conditions applied.

## 1. Introduction

The healthy properties of olive leaves (OL) are recognized in the traditional medicine and also supported by several scientific reports. The potential of olive leaves extracts to formulate functional ingredients and to obtain antioxidant and antimicrobial preservatives is promising [1,2]. Currently, in the phytopharmacy sector, olive leaves and fruits extracts are key ingredients of dietary supplements and nutraceuticals (infusions, capsules, liquid solutions, etc.) due to their cardiovascular health promoting properties, among other effects. Moreover, the use of synthetic hydroxytyrosol has been approved as a novel ingredient to be added to oils and spreadable fats [3], which is a precedent for using natural extracts containing this compound. Furthermore, Rodrigues et al. [4] also suggested that the bioactive compounds present in olive by-products, including antioxidants, can become a source of anti-aging or hydration active ingredients for cosmetics.

Hydroxytyrosol and their derivatives are some of the active components both to improve health, as several clinical trials suggest [5,6,7,8], and the oxidative stability of oils [9,10]. Nonetheless, the hydroxytyrosol cluster composition depends on the olive biomass type and the extraction conditions [11,12]. For example, olive leaves and olive leafy byproducts are richer in oleuropein, while olive fruits and its derived byproduct, olive pomace, contain more hydroxtyrosol, among other derivatives [12,13,14,15].

**Figure 1:**
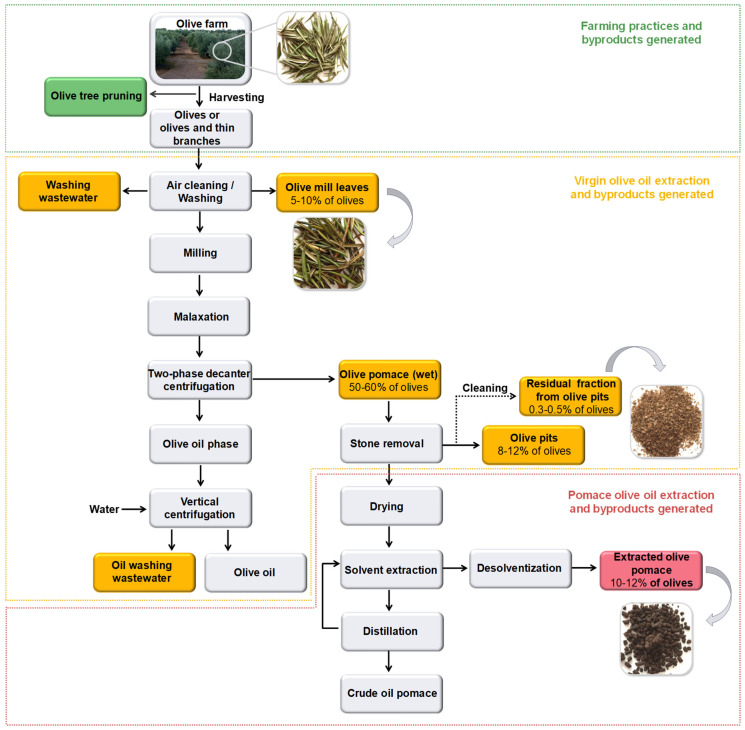
Simplified schemes of the extraction of virgin olive oil and pomace olive oil and the byproducts (squares in green, yellow, and pink) generated during the production steps.

In the olive pomace extracting industry, the extracted olive pomace (EOP) is obtained after the extraction of the residual oil contained in the olive pomace, generally, using hexane (Figure 1). This solid biomass is generated in high amounts; around 10–12% (*w*/*w*) of the olives processed in the mills. In Spain, the olive stones fragments are recovered from the olive pomace and sometimes cleaned by a pneumatic process to enhance their energetic potential (Figure 1). This residual fraction derived from the olive pits cleaning (RFOPC or residual pulp) consists of rests of olive pulp, mainly, skin crushed into fragments [11,16,17]. While crushed pits represent around 8–10% of olives weight, the average percentage of RFOPC in the latter fraction is up to 4% [17]. Interestingly, EOP and RFOPC can be produced in the same facility where the main products, olive oil and olive pomace oil, are produced, implying additional advantages for their valorization, i.e., reduced collection and transport costs. While olive pits and EOP are used as a relatively low-cost biofuel, the RFOPC has no current industrial application. Nonetheless, the application of the EOP as a biofuel has some constrains [18] and the removal of a part of the extractive fraction (non-structural components) could improve its energetic use [19]. In this regard, the extractive fraction contains valuable bioactive compounds, including phenolic compounds [11], and hence another alternative would be to obtain antioxidants from these cheap and abundant bioresources before applying other valorization strategies [19,20]. Therefore, their comprehensive characterization may give also clues about the phenolic composition, including the hydroxytyrosol cluster and the presence of other bioactive compounds.

In this context, to recover antioxidants like phenolic compounds from olive-derived byproducts, new trends included the use of ultrasound to assist the extraction process, favoring the mass transfer, shortening the extraction time and/or reducing the solvent necessities [14,21,22]. Nevertheless, the extraction of antioxidants generates a large residual fraction that is worthy of valorization since it can provide an extra income and move towards the circular bioeconomy. For this purpose, antioxidants can be obtained as a first step previous to a further fractionation of the rest of components present in the biomass [14,19,21]. Another alternative is to recover the antioxidants in the lateral streams obtained after the pretreatment of these biomasses, for example, for the conversion of the sugar fraction to biofuels. Nevertheless, it generally requires severe thermal treatments and more thermolabile bioactive compounds could be affected. For example, oleuropein seems to be resistant at least in part [23], but it depends on the conditions applied to the olive leafy biomass [21].

Therefore, in this work, an integrated scheme was applied to fractionate EOP and the new byproduct RFOPC and to characterize the fractions obtained for further valorization. This consisted of ultrasound-assisted extraction (UAE) as a first step to recover antioxidant extracts and an alkaline extraction as second step to fractionate the residual lignocelullosic fraction, according to Contreras et al. [14]. The phenolic composition of extracts obtained from EOP and RFOPC in the first step was characterized, including the hydroxytyrosol cluster, and the antioxidant activity measured, being compared to those extracts obtained from olive leafy biomasses. The second step enabled to recover a liquid fraction, whose composition was characterized in terms of protein, lignin, sugars, and antioxidant properties.

## 2. Materials and Methods

### 2.1. Reagents, Standards, and Samples

#### 2.1.1. Reagents and Standards

The following reagents were purchased from Sigma-Aldrich (St. Louis, MO, USA): Folin and Ciocalteu′s phenol reagent, sodium carbonate, 2,2′-azobis (2-methylpropionamidine) dihydrochloride (AAPH), 2,4,6-tris (2-pyridyl)-s-triazine (TPTZ), 2,2′-azino-bis(3-ethylbenzothiazoline-6-sulfonic acid) (ABTS) diammonium salt, 6-hydroxy-2,5,7,8-tetramethylchroman-2-carboxylic acid (Trolox), fluorescein sodium salt, potassium persulfate, and ferric sulfate. The following reagents were bought from PanReac AppliChem (Barcelona, Spain): dehydrated sodium phosphate, sodium acetate, ferric chloride, hydrochloric acid, ethanol, formic acid, glacial acetic acid, acetonitrile, and acetone. Sodium hydroxide was purchased from VWR Chemicals (Radnor, PA, USA).

Phenolic standards (degree of purity ≥98%, *w*/*w*) were obtained from Extrasynthese (Genay, France) (hydroxytyrosol and oleuropein) and Sigma-Aldrich (St. Louis, MO, USA) (gallic acid, rutin, and caffeic acid).

#### 2.1.2. Samples

EOP was obtained from the olive pomace extracting industry Oleocastellar S.A. (Castellar, Jaén, Spain) and RFOPC from Peláez Renovables (Jaén, Spain). The samples were ground using an Ultra Centrifugal Mill ZM 200 (1 mm sieve) (Retsch GmbH, Haan, Germany) before the determination of the chemical composition and extraction.

### 2.2. Determination of the Chemical Composition

The moisture and the content of extractives, lignin, carbohydrates, and ash of the samples were determined according to the standard National Renewable Energy Laboratory procedure [24]. Aqueous, ethanolic and hexane extractives were determined using Soxhlet extraction and gravimetric analysis. The characterization of carbohydrates and lignin was performed after acid hydrolysis. The liquid fraction was subjected to high-performance liquid chromatography (HPLC) analysis to quantify monomeric sugars and acid soluble lignin was determined spectrophotometrically at 205 nm. Acid insoluble lignin was determined by gravimetric analysis, taking into account the ash content in this fraction. Moreover, the crude protein content of the byproducts was determined by elemental analysis (TruSpec Micro, Leco, St. Joseph, MI, USA) using a conversion factor of 6.25. All analytical determinations were performed in triplicate.

### 2.3. Ultrasound-Assisted Extraction

The samples were mixed with ethanol/water (47:53, *v*/*v*) at a solid-to-liquid ratio of 6:100 (*w*/*v*, dry weight, d.w.) and sonicated in an ultrasonic bath (40 kHz) (Ultrasons, J.P. Selecta, Barcelona, Spain) for 50 min, according to our previous optimized method [14]. The mixture was centrifuged at 1717× *g* for 15 min, the supernatants were collected, and the recovered volume was measured. All the extractions were done in triplicate. Finally, a portion of the extracts was oven-dried (at 105 °C) till constant weight to estimate the total extraction yield, which was referred to the initial dry byproduct weight (%). Other portion was filtered with a syringe filter (nylon, 0.45 μm pore size) (SinerLab Group, Madrid, Spain) for further analysis.

### 2.4. Total Phenol Content and Antioxidant Capacity Assays

Total phenol content (TPC), Trolox equivalent antioxidant capacity (TEAC) and ferric ion reducing antioxidant power (FRAP) were determined using colorimetric assays in transparent microplates according to Medfai et al. [12]. Basically, these assays measure the ability to reduce the Folin and Ciocalteu′s phenol reagent, ABTS^●+^, Fe^3+^, respectively, which changes color when reduced and this change is correlated with the antioxidant concentration. For that, a Bio-Rad iMark^TM^ microplate absorbance reader was used (Hercules, CA, USA) at 750 nm (Folin-Ciocalteu and TEAC assays) and 595 nm (FRAP assay). Using standard curves (R^2^ > 0.99), the TPC results were expressed as gallic acid equivalents (GAE) (25 to 300 μg/mL) and the TEAC and FRAP results as Trolox equivalents (TE) (6 to 330 μM), respectively.

The oxygen radical absorbance capacity (ORAC) was performed using black microplates according to Medfai et al. [12]. Fluorescent measurements with excitation and emission wavelengths of 485 nm (±10) and 528 nm (±10), respectively, were obtained using a BioTek Synergy HT (Winooski, VT, USA) and acquired with Gen5 (BioTek) every min for 120 min. The data was normalized using the initial reading, the area under curve (AUC) for each well was estimated and the net AUC was calculated by subtracting the AUC corresponding to the blank. ORAC values were expressed as TE (standard curve from 0.5 to 20 µM; R^2^ > 0.99).

The filtered aqueous-ethanolic extracts (Section 2.4) and the alkaline extracts obtained according to next Section 2.7 were measured. The latter samples were neutralized, centrifuged, and filtered (0.45 μm nylon syringe filters) before analysis.

Caffeic acid was used as positive control, obtaining the following values: TEAC = 1.23 ± 0.09 mmol equivalents of Trolox; FRAP = 1.15 ± 0.05 mmol equivalents of Trolox; ORAC = 4.29 ± 0.31 mmol equivalents of Trolox. These values agreed with those reported previously [25].

### 2.5. HPLC-Mass Spectrometry (MS) and Diode Array Analyses

Reversed phase (RP)-HPLC-MS and -MS^2^ analyses were performed in an Agilent 1100 HPLC (Agilent Technologies, Waldbron, Germany) connected on-line to an ion trap (IT) via an electrospray interface (Esquire 6000; Bruker, Bremen, Germany), according to Medfai et al. [12]. Phenolic compounds were eluted at 0.35 mL/min using Milli-Q^®^ water and formic acid (0.1%, *v*/*v*) as solvent A and acetonitrile and formic acid (0.1%, *v*/*v*) as solvent B. A Kinetex core-shell C18 column (2.1 × 50 mm, 2.7 μm) (Phenomenex, Barcelona, Spain) and a linear gradient of solvent B in A were used: 4%, 0 min; 7%, 1 min; 30%, 15 min; 40%, 4.5 min; 100%, 4.5 min; 100%, 2 min; 4%, 1.5 min; and 4%, 7 min. The injection volume was 10 μL.

MS spectra were recorded over the mass-to-charge (*m*/*z*) range of 100–1200 in the negative ionization mode and 4 spectra were averaged. Auto MS/MS analyses were performed at 0.6 V and acquired in the aforementioned range. About 2 spectra were averaged in the MS/MS analyses. The data were processed using DataAnalysis (version 4.0) from Bruker.

In addition, analyses by HPLC (Agilent 1200) coupled to quadrupole-time-of-flight (QTOF)-MS and MS/MS (Agilent 6530B Accurate Mass Q-TOF) were performed to obtain high resolution mass data. The interface was an electrospray ionization source. The column and the gradient use were the same as before. The MS parameters were applied according to Ammar et al. [26] in auto-MS mode, with some modifications. The spectra were acquired in the negative ionization mode, over the *m*/*z* range 60–1200 Da. For accurate *m*/*z* measurement reference mass correction was performed with a continuous infusion of trifluoroacetic acid ammonium salt (*m*/*z* 112.9856) and hexakis 1H,1H,3H–tetrafluoropropoxy) phosphazine (*m*/*z* 1033.9881) (Agilent Technologies, Waldbron, Germany). MassHunter Qualitative Analysis B.06.00 (Agilent Technologies) was applied for data treatment to generate molecular formula with a mass accuracy limit of 5 ppm.

Hydroxytyrosol and oleuropein were quantified using external standard calibration by RP-HPLC-diode array detection at 280 nm, according to Contreras et al. [21]. The curves (*R*^2^ > 0.99) were *y* = 20395*x* − 15047 for hydroxytyrosol (1.25 to 500 mg/L) and *y* = 5591*x* + 11,911 for oleuropein (2.5 to 1000 mg/L).

### 2.6. Alkaline Extraction of the Residual Extraction Fraction and Determination of the Protein Content and Profile

Alkaline extraction was performed according to previous optimized conditions using a solid-to-liquid ratio of 1:10 (*w/v*) and sodium hydroxide 0.7 M and 0.4 M in a bath (JULABO GmbH, Seelbach, Germany) at 100 °C and 80 °C, respectively, for 240 min (at 150 rpm) [14]. After subsequent centrifugation, which was performed at 1717× *g* for 15 min (Herolab, Wiesloch, Germany), supernatants were collected for further analysis and the recovered volume measured. The solubilized protein in the alkaline extracts was determined using a Bradford kit assay from Bio-Rad (Irvine, CA, USA), with some modifications, and referred to a standard calibration curve of bovine serum albumin (BSA).

The protein extract was neutralized using HCl 2 M and proteins (100 μL) were precipitated with 400 μL of acetone at cold conditions for 20 min and followed by centrifugation at 10,000× *g* for 10 min. The protein pellets were dissolved in 50 μL of Laemmli sample buffer (with 2-mercaptoethanol at 5%, *v*/*v*). The separation was performed on Mini-PROTEAN^®^ TGX ™ Precast Gels (Bio-Rad, Irvine, CA, USA) at 200 V in a Mini-PROTEAN^®^ tetra cell (Bio-Rad, Irvine, CA, USA) and using Tris/Glycine/SDS buffer (Bio-Rad, Irvine, CA, USA) as running buffer. Gels were stained using Coomassie Brilliant Blue R-250 (Bio-Rad, Irvine, CA, USA) for 90 min and destained overnight.

### 2.7. Sugars Analysis and Lignin Determination in the Alkaline Extracts

Samples were acidified around 3.5 by adding HCl 2 M and centrifuged (10,000× *g* for 10 min) (MicroCen 16, Herolab GmbH Laborgeräte, Wiesloch, Germany). Then, the supernatants were collected and filtered (0.45 μm nylon filters). Additionally, oligomeric sugars were measured upon acid hydrolysis using sulfuric acid at 120 °C for 30 min. All samples were measured using HPLC with a refractive index detector and an ICSep ICE-COREGEL-87H3 column (Transgenomic, Inc., Omaha, NE, USA) [27].

Soluble lignin concentration was estimated in alkaline extracts after centrifugation (10,000× *g* for 10 min) according to Guerra [28] at 0.1 M NaOH as: A/(ε × l), where A is the absorbance at 280 nm and ε = 9.7 L/g cm. The measurements were performed by an UV-Vis spectrophotometer (UV-1800, Shimadzu Schweiz GmbH, Reinach BL, Switzerland) with a 1 cm quartz cuvette. The spectra were also recorded from 190 to 800 nm, which were processed by UVProbe 2.32 (Shimadzu Schweiz GmbH). The first derivative spectra were obtained using the latter software and applying a δ λ of 20.

### 2.8. Statistical Analysis

Data are expressed as mean ± standard deviation of three analyses. One-way analysis of variance (ANOVA) and LSD for multiple comparisons were performed using Statgraphics Centurion XVII (StatPoint Technologies, Inc., Warrenton, VA, USA). Pearson correlation was performed using Microsoft Excel 2007 (Redmond, WA, USA) and Statgraphics Centurion XVII.

## 3. Results and Discussion

### 3.1. Chemical Composition

The chemical composition and the elemental analysis results of the studied byproducts are shown in Table 1 and compared to olive leafy biomass. Although both byproducts, EOP and RFOPC, contain olive fruit rests, the chemical composition was quite different. Nevertheless, as in OL the major fractions were aqueous-ethanolic extractives (non-structural components), particularly, in EOP (52.85%), and lignin, particularly, in RFOPC (32.25%). The highest content of cellulose and hemicellulose was found in RFOPC (around 27.3%), while it contained the lowest protein amount (4.50%) compared to EOP (9.36%) and leaves (up to 9.34%). Moreover, EOP has a high content in ash (10.06%) as OML.

In general, these results agreed well with previous studies on EOP [20,30], but there is little information about RFOPC, as far as we know. It remarks that besides olive leafy biomasses, EOP and RFOPC contain a high amount of extractives, including free and oligomeric sugars and the sugar alcohol mannitol, which is a marketable sweetener and a drug [11]. In this regard, the content of mannitol was only remarkable in EOP (5.36% with respect to the extractives content), even higher than in leaves. Moreover, the extractive fraction is interesting since it presents olive bioactive components, as next sections highlight.

### 3.2. Extraction of Antioxidants by UAE as a First Valorization Step

#### 3.2.1. Yield, Total Phenolic Content, and Antioxidant Characteristics of the Extracts

The aforementioned results suggest that the extractive fraction is worth of study because of its high content. Therefore, UAE was applied to recover antioxidant extracts from EOP and RFOPC using an aqueous ethanol solution as a first valorization step, according to Contreras et al. [14]. This method was selected to evaluate how the biomass type affects and thus olive leafy biomasses, EOP and RFOPC, were compared (Table 2).

EOP provided the highest total extraction yield by UAE and its capacity to retain solvent was low, indicating that the technical and theoretical yields for these parameters will be similar. Moreover, the solubilization of antioxidants, expressed in terms of biomass weight, from EOP was higher than that for OL and OML, while RFOPC showed the lowest values (Table 2). All these data showed good correlation values with the extractives content (*r* > 0.958), the total phenolic content and between each other (*r* > 0.914) (Table S1). This suggests that phenolic compounds are the main antioxidant compounds in the extracts.

Nonetheless, in terms of purity, the antioxidants extracts of RFOPC only showed a slightly lower potency than the former byproducts. It is explained by the fact that a lower amount of solids were released from RFOPC (Table 2), which can be correlated with its lower amount of extractives (Table 1).

As for olive leafy biomasses, EOP and RFOPC antioxidants showed a higher efficiency for scavenging peroxyl radicals by hydrogen atom transfer mechanisms (ORAC) than reduction properties by electron transfer mechanisms (TEAC and FRAP) under the conditions assayed [12]. Therefore, the antioxidants from these byproducts could have some similarities, being probably relevant to scavenge radicals in vivo and in food systems and further work is necessary. Moreover, the chemical characterization of the extracts is necessary to reveal information about the hydroxytyrosol cluster.

#### 3.2.2. Characterization of the Antioxidant Extracts by HPLC-MS Analyses

##### Phenolic Compounds

**Figure 2:**
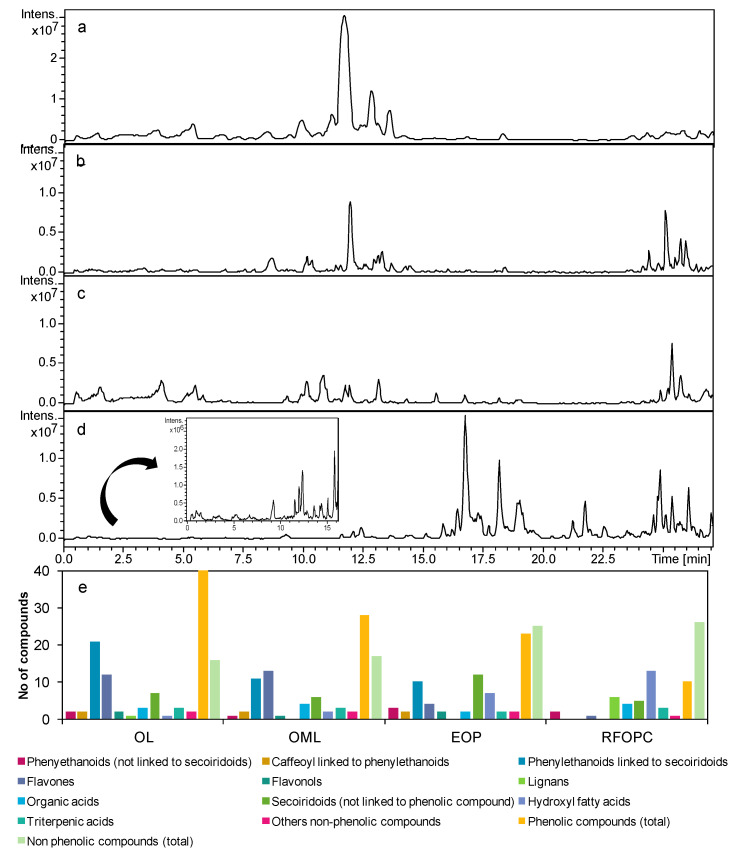
Base peak chromatograms of (**a**) olive leaves, (**b**) olive mill leaves, (**c**) exhausted olive pomace, and (**d**) residual fraction from olive pit cleaning obtained by RP-HPLC-IT-MS. (**e**) Number of compounds characterized per chemical class. OL: olive leaves; OML: olive mill leaves; EOP: extracted olive pomace; RFOPC: residual fraction from olive pit cleaning.

Two MS analyzers, an IT and a QTOF, were applied to get maximum information about the phenolic class and other phytochemicals present in the antioxidant extracts. Both have demonstrated multiclass potential to characterize phenolic compounds and other bioactives present in olive-derived biomasses [26,31]. The former enabled us to compare the RP-HPLC-MS profiles and some phenolic compounds with those characterized in our previous work on OL and OML [12,14]. The second one provided mass accurate measurements for structural confirmation and characterization of novel compounds [26,32]. Figure 2 depicts the MS profiles obtained by RP-HPLC-IT-MS of OL, OML, EOP, and RFOPC after UAE extraction. It shows that all samples show qualitative differences, especially those derived from olive pomace, which were also different between each other.

Then, the phenolic compounds were characterized by HPLC-MS and -MS/MS based on their accurate *m*/*z* value, molecular formula, and mass fragmentation patterns, which were compared to those in an in-source library and literature [12,26,33,34,35]. A total of 55 phenolic compounds were characterized in the four extract types and belonged to several phenolic classes, i.e., phenylethanoids (3), caffeoyl phenylethanoid derivatives (2), caffeoyl derivatives (2), phenyl ethanoids linked to secoiridoids (including oleuropein) (26), flavones (13), flavonols (2), and lignans derivatives (7) (Table 3). The number of compounds found in each extract type is shown in Figure 2e. OL was qualitatively richer in phenolic compounds than the rest of the samples, followed by OML. Not surprisingly, OML and OL shared most phenolic compounds since OML is composed of olive leaves and thin branches. Nonetheless, quercetin glucoside and some oleuropein derivatives were only present in OL; particularly, the novel derivatives 49, 50, 52–54. These compounds were characterized in our previous work [12], but here the QTOF analysis enabled us to establish their molecular formulae and confirm the presence of fragments related to oleuropein. On the basis of the MS information, it seems that they can be formed by the conjugation of oleuropein and hydroxycinnamic acids (compounds 46 and 49) and fatty acids (compounds 50, 52–54) (Table 3), but further nuclear magnetic resonance analysis is required for confirmation.

The EOP contained mainly hydroxytyrosol derivatives (Table 3), but the hydroxytyrosol cluster was different to that of OML and OL. It presented mainly free forms (hydroxytyrosol, hydroxytyrosol glucoside and tyrosol glucoside), hydroxytyrosol/tyrosol linked to secoiridoids, including oleuropein, ligustroside, and 3,4-DHPEA-EDA (dialdehydic form of elenolic acid linked to hydroxytyrosol), and to caffeic acid (verbascoside and isoverbascoside). Some of these compounds and the caffeoyl derivatives, caffeoyl- and *p*-coumaroyl-6′-secologanoside, have been found in olive pomace [35,36], and hence it remarks that they resist, at least in part, the processing of olive pomace to obtain pomace olive oil and EOP. Moreover, the hydroxytyrosol cluster included two novel hydroxytyrosol derivatives (compound 12 and 51). Compound 12 seems to be a glycosylated derivative of 3,4-DHPEA-EDA and compound 51 was formed by the linking between hydroxytyrosol and desoxy elenolic acid, whose molecular ion was found in the MS/MS spectrum and some product ions, e.g., *m*/*z* 181 derives from the loss of CO_2_. This derivative of elenolic acid is present in olive oil [37].

**Figure 3:**
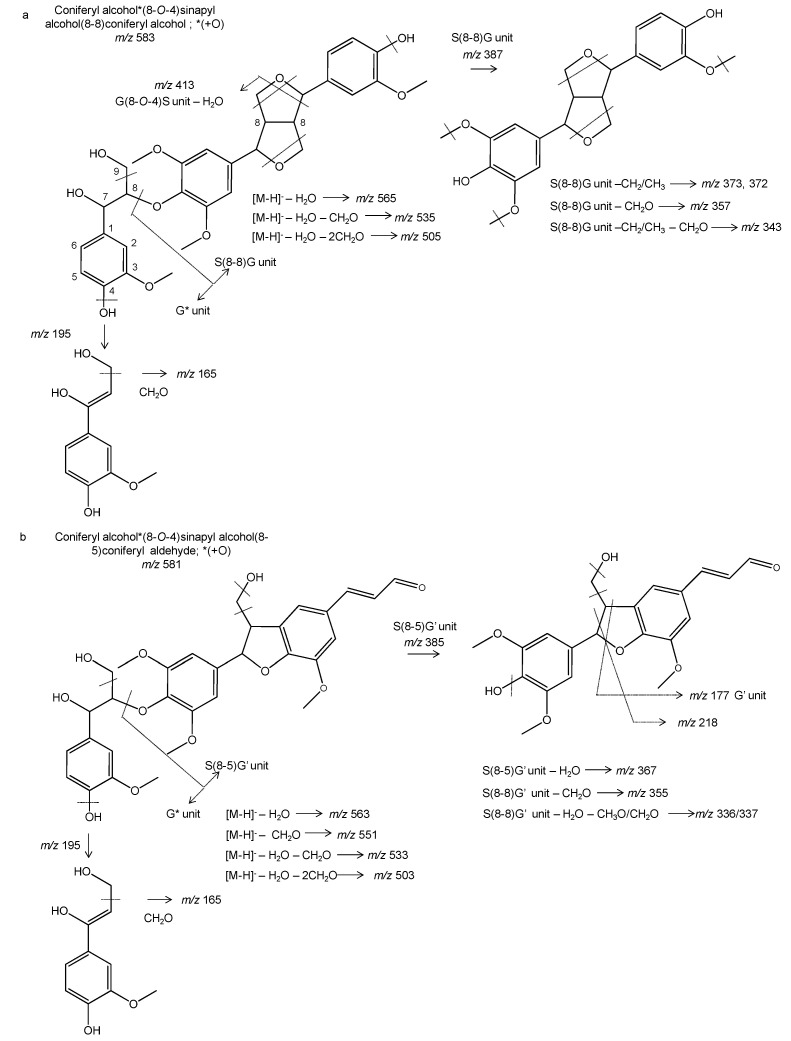
Tentative structure of novel free trilignols, (**a**) at *m*/*z* 583, (**b**) *m*/*z* 581 and derivatives characterized by mass spectrometry in olive byproducts.

RFOPC also contained some of the latter compounds, including hydroxytyrosol, but its phenolic profile was quite different. Interestingly, six novel phenolic compounds were found in RFOPC, i.e., trilignols (Figure 3). These compounds have previously been characterized in wild-type poplar (*Populus tremula* × *Populus tremuloides*) xylem as lignin oligomers [33] and in root exudates of *Arabidopsis thaliana* [38], but not in olive matrices. Basically, the phenolic compound at *m*/*z* 583 consists of coniferyl and sinapyl alcohol moieties linked by 8-8 linkage unit, where the syringyl moiety is linked to coniferyl alcohol via 8-*O*-4 linkage unit, which is hydroxylated in the position 7 (Figure 3a). Their fragmentation pattern is characterized by the presence of an ion at *m*/*z* 373, which is characteristic of X(8-8)X-containing trilignols [33]. Moreover, the fragmentation at the 8-*O*-4 linkage gave two main product ions, i.e., *m*/*z* 387 which correspond to the dilignol formed by coniferyl and sinapyl alcohols, and the counterpart *m*/*z* 195 (hydroxylated coniferyl alcohol). The other trilignols (*m*/*z* 581) is similar to the former, but it contains two fewer hydrogens in their structure. This indicated that the coniferyl alcohol was substituted by a coniferyl aldehyde moiety, which implies to connect via 8-5 linkage. This was characterized by the presence of the odd ion *m*/*z* 218 (C_12_H_10_O_4_^−^) that derive from the fragmentation of the phenylcoumaran (8-5) linkage. Furthermore, some potential degradation products could be detected at *m*/*z* 367 and *m*/*z* 337, sharing a similar structure to those fragments found at the same *m*/*z* values found in both IT- and QTOF-MS/MS spectra (Figure 3b). Both compounds showed the presence of an ion at *m*/*z* 177 with the molecular formula C_10_H_9_O_3_, which correspond to coniferyl aldehyde, reaffirming their structures. Similarly, the compound at *m*/*z* 569 presented this fragment in MS/MS spectrum. Other fragments were at *m*/*z* 393 and 373, which could be hydroxylated coniferyl and sinapyl alcohols moieties (-C) and the latter linked to coniferyl aldehyde (-C). This indicates that the carbon loss occurs at the sinapyl moiety when compared to the compound at *m*/*z* 581.

**Figure 4:**
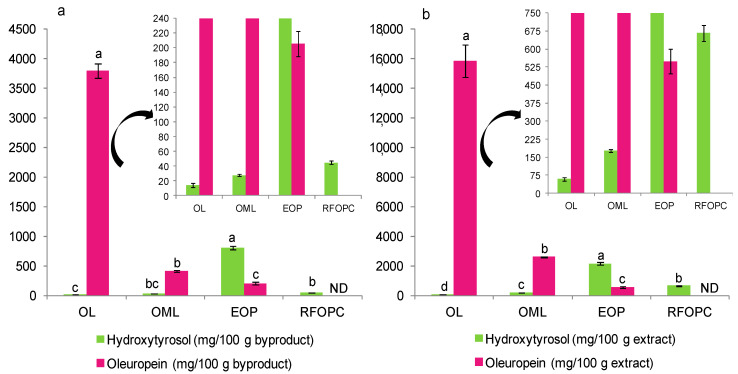
Content of hydroxytyrosol and oleuropein in olive byproducts in terms of: (**a**) Byproduct weight (d.w.) and (**b**) extract weight (d.w.). For each compound, different lowercase letters indicate significant differences between the samples (*p* < 0.05). ND, not detected.

Finally, the content of hydroxytyrosol and oleuropein was determined as a way to standardize and compare the extracts with those obtained in previous studies since both are reference olive phenolic compounds due to their biological properties, as commented in the introduction. The content of oleuropein was higher in OL followed by OML and EOP, both in terms of biomass and extract weight (Figure 4). Alternatively, EOP was the richest byproduct in hydroxytyrosol (0.8 g/100 g biomass weight and 2.1 g/100 g extract weight) followed by RFOPC. The values in EOP are higher than those reported in olive pomace [15,39] and other pomace byproducts [35,40] obtained by different technologies, including UAE and pressurized liquid extraction. Therefore, this work highlights that EOP is a source of hydroxytyrosol and its derivatives, which can be extracted before the use of EOP as a biofuel, for example.

##### Non-Phenolic Compounds

**Figure 5:**
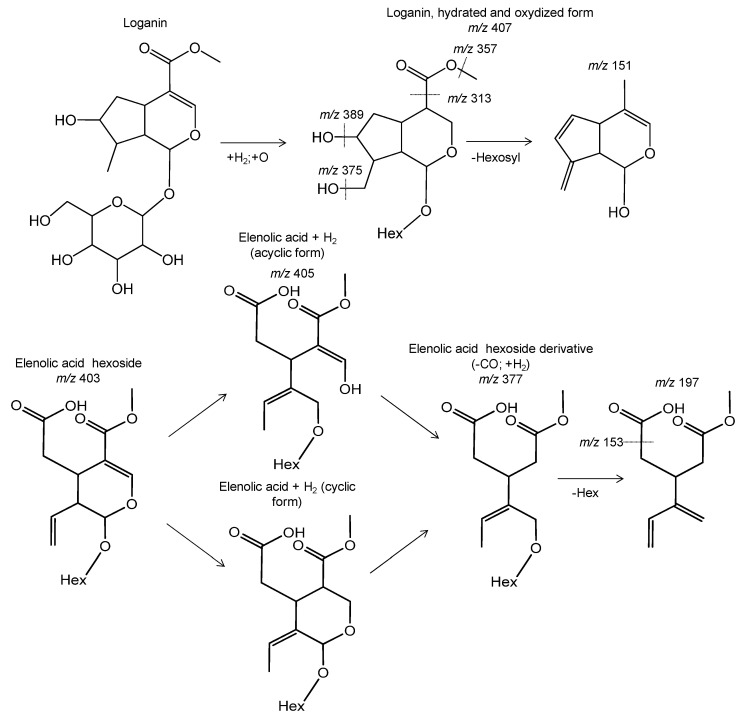
Tentative structure of novel free secoiridoids characterized by mass spectrometry in olive byproducts.

Table 4 shows the MS information of other compounds found in the extracts. In addition to the phenolic compounds, free secoiridoids, i.e., not linked to a phenolic moiety, were characterized including oleoside, secologanoside, elenolic acid hexosides and hydroxyelenolic acid, as well as six novel structures, which were proposed according to the MS information (Figure 5). Cyclic and acyclic forms are provided because it cannot be confirmed by MS what form could be or whether these forms coexist.

Other compounds detected were mannitol and some organic acids, a jasmonic acid derivative, hydroxyl fatty acids, and triterpenic acids (Table 4; Figure 2e). Their fragmentation patterns agreed well with previous studies [26,38,41,42,43].

Among them, hydroxy fatty acids, a type of oxylipins, are bioactive metabolites derived from the oxygenation of polyunsaturated fatty acids. They can be applied as starting materials for the synthesis of polymers and as additives for the manufacture of lubricants, emulsifiers, and stabilizers [44]. In plants, these compounds are formed after the release of free fatty acids from triglycerides due to the effect of lipolytic enzymes [42]. They forms part of cutin; a polymer of C16 and C18 fatty acids, with one or more hydroxy groups or epoxides, held together mainly by primary alcohol ester linkages [45]. Since cutin is present in fruit peels [46], it explains their presence in RFOPC, which is rich in olive skin [16,17]. In EOP, some of these compounds were also present. Moreover, although some of these compounds have been characterized in olive samples, the MS data revealed the presence of dimeric structures, which have not been reported before. Their *m*/*z* values correspond to the sum of the monomeric forms with a loss of water due to their linkage.

Regarding triterpenic acids, this class was composed of oleanolic, maslinic acid, pomolic acid, and hydroxylated maslinic acid. With the exception of the latter compound, oleanolic and maslinic acids have been reported before in OL, OML, and RFOPC [16,47]. Alternatively, hydroxylated maslinic acid has recently been characterized in olive oil and olive flour [37]. This is interesting since besides the presence of antioxidants, these compounds could give an extra value to the extracts due to their prominent bioactive properties, including cardioprotective properties [48].

### 3.3. Second Extraction Step Using Akaline Conditions: Evaluation of the Solubilization of Proteins, Sugars and Lignin

#### 3.3.1. Solubilized Protein

Alkaline extraction can promote the solubilization of proteins, which can be purified for different applications in the food and other sectors [49,50]. Then, two conditions were tested to evaluate how the biomass type and alkaline extraction conditions affect the solubilization of proteins and the recovery values as a second valorization step: (i) 0.4 M NaOH, as solubilization agent, at 80 °C; and (ii) 0.7 M NaOH, as solubilization agent, at 100 °C [14,21]. The protein content varied from 3.7 g/100 g byproduct (RFOPC) to 6 g/100 g byproduct (EOP), i.e., a recovery between 49% (OML) and 100% (RFOPC) (Table 5). These recovery values for OL, OML and EOP are in the range of other agricultural and agro-industrial resources extracted using alkaline solutions [11,49], while RFOPC showed superior values as shown Zhang et al. [51] for tea leafy residue. Although EOP and RFOPC derive from the same source, olive pomace, the recovery values were different. Moreover, regardless of the applied conditions, the recovery was similar for EOP (around 60%), while for RFOPC the maximum value was achieved using strong alkaline-thermal conditions. The feedstock composition can affect the protein extractability as shown Sari et al. [52]. The latter authors suggested that cellulose and oil are the main constituents that hamper the extractability of proteins, but RFOPC has the highest value for both components. Hence, other factors can affect the separation of proteins, maybe, to a higher interaction with the lignocellulosic matrix, as commented on next.

**Figure 6:**
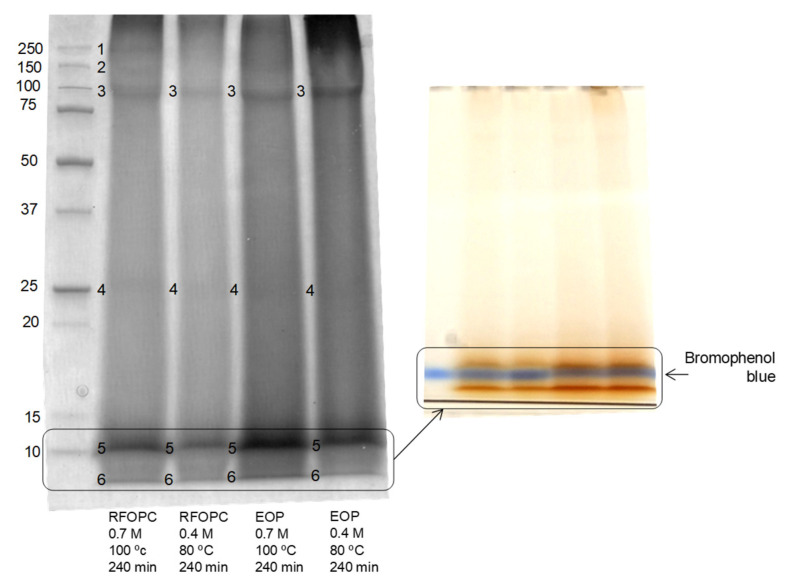
SDS-PAGE profiles of protein products obtained from the residual fraction from olive pit cleaning (RFOPC) (lanes 2 and 3) and extracted olive pomace (EOP) (lanes 4 and 5) by alkaline conditions after phenolic extraction. The protein markers kit was at lane 1.

Concerning the protein profile (Figure 6), all extracts contained protein bands around 100 kDa (band 3), 25 kDa (band 4) and 10 kDa (band 5), but the second one was very light in EOP. This band could be related to oleosins, which are alkaline proteins with molecular masses ranging between 15 and 26 kDa [53]. Moreover, lipoxygenase in olive fruit has a molecular weight around of 98 kDa [54]. Other band (band 6) with a molecular weight lower than bromophenol blue (0.67 kDa) was also observed, indicating the potential hydrolysis of proteins, as other studies on agri-food proteins suggested when using alkaline conditions for solubilization [11]. In fact, this and the 10 kDa band were also observed in OL after alkaline extraction [14,21]. They had a characteristic brown color. Among them, a protein/peptide around 10 kDa has also been detected in olive seeds [55]. Alternatively, two bands around 150 kDa and 250 kDa were detected in RFOPC when using strong alkaline conditions. Vioque et al. [56] suggested that olive pomace proteins can be highly denatured and/or associated with the fiber and other components and maybe the different processing that EOP and RFOCP are subjected to could affect these interactions or their solubilization in a different way, explaining their different solubility.

#### 3.3.2. Sugars, Sugar Alcohols, and Lignin

Alkaline treatments can also break the lignocellulosic matrix for further valorization of the polymeric sugars. It can promote the release of a part of the hemicellulosic sugars and lignin to obtain a solid fraction enriched in cellulose, which can be applied to obtain sugars and valuable sugars derivatives (bioethanol, organic acids, etc.) [11,57,58].

**Figure 7:**
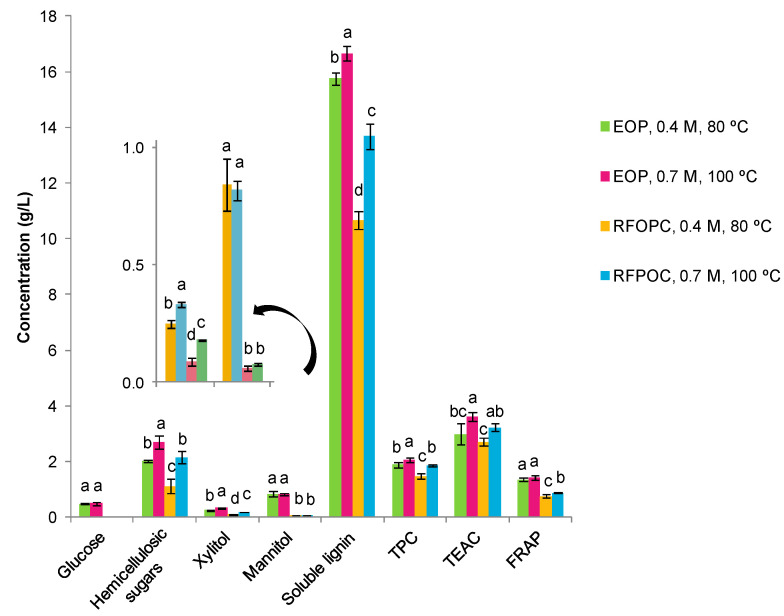
Content of sugar, sugar alcohols, and soluble lignin (g/L), total phenolic content (g gallic acid equivalents/L) and antioxidant activity (g Trolox equivalents/L) of the alkaline extracts obtained from the extracted olive pomace (EOP) and the residual fraction from olive pit cleaning (RFOPC). For each component, different lowercase letters indicate significant differences between the samples (*p* < 0.05).

In this regard, glucose was absent in alkaline extracts from RFOCP, while its content was around 0.5 g/L in the EOP extracts. This suggests that the dissolution of the cellulose was negligible or poor under the conditions tested. Alternatively, the content of hemicellulosic sugars increased up to 2.7 g/L when stronger alkaline-thermal conditions were applied (Figure 7). These values were lower than those found in OML [21], indicating that the fractionation results depend on the biomass. Moreover, the released sugars were mainly in the form of dimers/oligomers (92–100%). Regarding sugar alcohols, a part of the mannitol in EOP was also extracted (around 0.7 g/L) (Figure 6), while other part is present in the antioxidant extracts, as commented before. The content was in the range of that found in OL after similar alkaline conditions [14], remarking again that EOP is a good source of this sugar alcohol.

The content of soluble lignin was also estimated since, as commented before, alkaline treatments can lead to a partial delignification [59]. Soluble lignin ranged from around 10.7 g/L to 16.7 g/L (Figure 7). In both cases, the major content was found in EOP extracts. Moreover, the UV spectra of the extracts were measured and show the main characteristic absorption bands for lignin, but the profiles presented some differences (Figure S1). This difference was more evidenced when applying the first derivative (Figure S1). In angiosperms, lignin is mainly composed of coniferyl alcohol and sinapyl alcohol-derived units in varying ratios that depend on the biomass type [60]. Thus, this is expected that the phenolic units are, at least in part, preserved and released after the alkaline treatment, being able to react with the Folin and Ciocalteu′s phenol reagent. However, the biomass type and the treatment can modify the aromatic structures [61,62], explaining the differences found between the samples. In fact, the TPC values ranged from 1.47 g GAE/L to 2.06 g GAE/L, i.e., a solubilized amount ranging from 1.62 g GAE/100 g to 2.20 g GAE/100 g of byproduct (Figure 7), and it was correlated with the lignin content (*r* = 0.960). It also correlated with the antioxidant activities (*r* > 0.784) of these extracts. The highest values were observed for EOP. Other authors have shown that lignin has antioxidant properties and thereby it could serve as a natural source of antioxidants to replace synthetic ones [62]. Thus, this procedure is another way to obtain antioxidant extracts with solubilized lignin and particularly to valorize the EOP and the RFOPC residual fractions obtained after the first extraction step by UAE. The antioxidant activity was lower or in the range of the phenolic extracts obtained by UAE in the first valorization step, with an antioxidant activity ranging from 12.8 mmol TE/100 g to 17.2 mmol TE/100 g of byproduct in the TEAC assay and 3.6 mmol TE/100 g to 6.9 mmol TE/100 g of byproduct in the FRAP assay.

Overall, further studies are required to separate these components, mainly, protein and lignin, which can find application in different sectors, including the food and feed sectors. Alternatively, the solid fraction with the rest of polymeric sugars, specially, glucose can be converted into biofuels or other building blocks derivatives within a biorefinery framework.

## 4. Conclusions

As a first step of valorization, UAE can be successfully applied to extract phenolic compounds from olive pomace-derived byproducts using aqueous-ethanol, particularly, EOP and RFOPC. The solubilization, extract richness and phenolic composition depended on the bioresource used. Among other phenolic compounds, EOP was a source of hydroxytyrosol and its derivatives: hydroxytyrosol glucoside, tyrosol glucoside, oleuropein, ligustroside, 3,4-DHPEA-EDA and its derivative, hydroxytyrosol linked to desoxy elenolic acid, verbascoside, and isoverbascoside. Alternatively, RFOPC resulted to be a source of novel trilignols. All of these extracts had good antioxidant properties compared to OL and OML extracts. Other compounds were present in the extracts, including free secoiridoids (i.e., not linked to phenolic compounds) and bioactive compounds such as triterpenic acids, while hydroxyl fatty acids were mainly present in RFOPC.

When the residual fraction obtained after UAE was subjected to an alkaline treatment for fractionation, the liquid fraction was rich in protein and it also contained soluble lignin, which conferred antioxidant properties to the extract. Due the selective release of protein and lignin in the liquid fraction, the recovery of a solid fraction rich in polymeric sugars is expected for further applications in biorefinery such as biofuel production. Overall, these results can be useful in a more sustainable olive sector promoting biorefinery approaches.

## Figures and Tables

**Table 1:** Chemical composition of olive leafy and pomace-derived biomasses.

Component (%)	OL ^4^	OML ^4^	EOP	RFOPC
Chemical characterization				
Protein	9.34 ± 0.35	8.10 ± 0.38	9.36 ± 0.44	4.50 ± 0.33
Glucans ^1^	6.98 ± 0.13	9.89 ± 0.57	6.96 ± 0.35	12.23 ± 0.96
Glucose	7.68 ± 0.14	10.88 ± 0.62	7.65 ± 0.38	13.45 ± 1.06
Hemicellulose ^2^	5.69 ± 0.11	7.90 ± 0.18	8.17 ± 0.20	14.22 ± 0.99
Galactose	1.41 ± 0.07	1.58 ± 0.07	0.38 ± 0.02	0.48 ± 0.03
Mannose	0.60 ± 0.09	0.14 ± 0.01	ND	ND
Xylose	1.30 ± 0.10	4.57 ± 0.14	8.60 ± 0.23	15.20 ± 1.09
Arabinose	3.06 ± 0.06	2.59 ± 0.12	0.23 ± 0.02	0.37 ± 0.02
Acid soluble lignin	3.43 ± 0.06	2.58 ± 0.026	1.65 ± 0.011	1.46 ± 0.11
Acid insoluble lignin	12.66 ± 0.75	20.75 ± 0.43	19.22 ± 1.63	30.79 ± 2.51
Ash	5.07 ± 0.07	10.15 ± 0.10	10.06 ± 0.40	2.99 ± 0.02
Extractives	45.07 ± 1.49	35.77 ± 1.29	52.85 ± 0.72	26.29 ± 4.04
Aqueous	29.46 ± 0.34	23.08 ± 1.78	48.71 ± 0.54	8.39 ± 0.64
Ethanolic	15.61 ± 1.16	12.69 ± 0.51	4.14 ± 0.40	17.91 ± 3.48
Monomeric sugars ^3^	8.79	2.86 ± 0.13	5.58 ± 0.04	1.54 ± 0.15
Oligomeric sugars ^3^		8.46 ± 0.59	5.86 ± 0.68	10.27 ± 1.09
Glucose ^3^	7.33 ± 0.17	7.61 ± 0.60	7.90 ± 0.36	1.31 ± 0.43
Galactose ^3^	0.94 ± 0.03	1.36 ± 0.09	1.19 ± 0.07	1.93 ± 0.19
Mannose ^3^	ND	0.03 ± 0.02	0.42 ± 0.03	0.46 ± 0.07
Xylose ^3^	0.11 ± 0.01	0.31 ± 0.13	0.29 ± 0.26	4.97 ± 0.35
Arabinose ^3^	0.41 ± 0.04	2.00 ± 0.44	1.64 ± 0.08	3.13 ± 0.34
Mannitol ^3^	4.64 ± 0.23	2.63 ± 0.13	5.36 ± 0.07	0.64 ± 0.19
Fat	ND	9.85 ± 0.10	2.47 ± 0.34	8.72 ± 0.96
Acetyl groups	0.66 ± 0.01	0.77 ± 0.02	0.79 ± 0.59	1.97 ± 0.06
Elemental analysis				
N	1.49 ± 0.06	1.30 ± 0.06	1.50 ± 0.07	0.72 ± 0.05
C	48.38 ± 0.48	48.08 ± 0.66	49.65 ± 0.85	56.86 ± 1.24
H	6.48 ± 0.15	6.49 ± 0.08	6.23 ± 0.33	7.26 ± 0.40

^1^ As glucose. ^2^ As hemicellulosic sugars. ^3^ With respect to aqueous extractives. ^4^ Results from previous studies [14,29]. ND, not determined; OL: olive leaves; OML: olive mill leaves; EOP: extracted olive pomace; RFOPC: residual fraction from olive pit cleaning.

**Table 2:** Ratio of extracted volume (%), total extraction yield (%), total phenolic content (g of gallic acid equivalents/100 g), and antioxidant activity (mmol Trolox equivalents/100 g).

Biomass	Extracted Volume/Total Volume	Total Extraction Yield	Solubilization ^1^				Extract Richness ^2^			
			TPC ^3^	TEAC ^3^	FRAP ^3^	ORAC ^3^	TPC ^3^	TEAC ^3^	FRAP ^3^	ORAC ^3^
OL ^4^	83.33 ± 1.44 ^c^	24.00 ± 1.99 ^b^	3.33 ± 0.42 ^b^	11.03 ± 0.20 ^b^	14.24 ± 0.78 ^b^	72.69 ± 3.65 ^b^	14.03 ± 2.99 ^a^	46.18 ± 4.44 ^b^	59.53 ± 4.48 ^a^	272.77 ± 33.35 ^a^
OML ^4^	83.33 ± 0.00 ^c^	15.77 ± 0.88 ^c^	2.06 ± 0.06 ^c^	11.94 ± 2.96 ^b^	ND	ND	13.11 ± 0.68 ^a^	74.72 ± 19.33 ^a^	ND	ND
EOP	92.17 ± 0.00 ^a^	37.44 ± 0.38 ^a^	4.45 ± 0.09 ^a^	25.25 ± 0.20 ^a^	20.30 ± 1.28 ^a^	95.30 ± 5.93 ^a^	11.90 ± 0.30 ^a^	67.43 ± 1.08 ^a^	54.23 ± 3.62 ^a^	254.44 ± 13.78 ^a^
RFOPC	85.80 ± 0.25 ^b^	6.70 ± 0.33 ^d^	0.46 ± 0.02 ^d^	2.61 ± 0.31 ^c^	1.35 ± 0.16 ^c^	11.50 ± 0.87 ^c^	6.92 ± 0.33 ^b^	39.14 ± 6.09 ^b^	20.10 ± 1.83 ^b^	171.78 ± 12.98 ^b^

Different lowercase letters within a row indicate significant differences between the samples (*p* < 0.05). OL, olive leaves; OML, olive mill leaves; EOP, exhausted olive pomace; RFOPC, residual fraction from olive pits cleaning. ND, not determined. ^1^ Data expressed in terms of byproduct weight on dry basis. ^2^ Data expressed in terms of extract weight on dry basis. ^3^ EOP, extracted olive pomace; FRAP, ferric reducing antioxidant power assay; TEAC, Trolox equivalent antioxidant capacity; TPC, total phenolic content; OML, olive mill leaves; OL, olive leaves; ORAC, oxygen radical absorbance capacity; RFOPC, residual fraction from olive pit cleaning. ^4^ Results from previous studies [12,14].

**Table 3:** Phenolic compounds characterized in olive leaves (OL), olive mill leaves (OML), exhausted olive pomace (EOP), and residual fraction from olive pit cleaning (RFOPC).

N°	RT ^1^ (Min)	Molecular Formula ^2^	*m*/*z*^2,3^	Error (Ppm) ^3^	Score ^2^	MS/MS ^1^	Compound	Class	OL	OML	EOP	RFOPC
1	1.2	C_14_H_20_O_8_	315.11	−3.4	94	153, 135, 123	Hydroxytyrosol glucoside	Phenylethanoids (not linked to secoiridoids)	+	−	+	+
2	1.1	C_8_H_10_O_3_	153.06	1.1	99	123	Hydroxytyrosol	Phenylethanoids (not linked to secoiridoids)	+	+	+	+
3	1.9	C_14_H_20_O_7_	299.11	0.5	95	179, 161, 119, 101	Tyrosol glucoside	Phenylethanoids (not linked to secoiridoids)	−	−	+	−
4	7.3	C_27_H_30_O_15_	593.15	−2.8	94	503, 473, 383, 353	Apigenin 6,8-di-*C*-glucoside	Flavones	+	+	−	−
5	8.1	C_27_H_30_O_16_	609.15	−3.2	90	447, 285	Luteolin di-hexoxide 1	Flavone	+	+	−	−
6	8.4	C_25_H_32_O_14_	555.17	−3.5	91	537, 403, 323, 223	Hydroxyoleuropein 1	Phenylethanoids (linked to secoiridoids)	+	+	−	−
7	8.4	C_27_H_30_O_16_	609.15	−2.7	94	447, 285	Luteolin di-hexoside 2	Flavone	+	+	−	−
8	8.5	C_24_H_30_O_13_	525.16	−3.4	91	481, 389, 319, 195, 165	Demethyloleuropein	Phenylethanoids (linked to secoiridoids)	+	+	−	−
9	8.9	C_25_H_32_O_15_	571.17	−3.5	92	523, 403, 359, 223, 179	Dihydroxyoleuropein	Phenylethanoids (linked to secoiridoids)	+	−	−	−
10	9.2	C_27_H_30_O_16_	609.15	−3.5	91	447, 285	Luteolin di-hexoxide 3	Flavones	+	+	−	−
11	9.3	C_25_H_32_O_14_	555.17	−3.1	92	537, 403, 371, 323, 223	Hydroxyoleuropein 2	Phenylethanoids (linked to secoiridoids)	+	+	−	−
**12**	**9.3**	**C_23_H_32_O_11_**	**483.19**	**−3.5**	**93**	**347, 123**	**3,4-DHPEA-EDA derivative (+ hexose + H_2_)**	Phenylethanoids (linked to secoiridoids)	−	−	**+**	−
13	9.7	C_20_H_32_O_12_	463.18	−3.0	95	347, 301	Quercetin glucoside	Flavonols	+	−	+	−
14	9.9	C_27_H_30_O_16_	609.15	−3.2	92	447, 301, 179	Quercetin 3-*O*-rutinoside	Flavonols	+	+	+	−
15	9.9	C_29_H_36_O_15_	623.20	−3.3	91	461, 315	Verbascoside	Caffeoyl phenylethanoid derivatives	+	+	+	−
16	10.0	C_21_H_20_O_11_	447.10	−3.7	91	285	Luteolin 7-*O*-glucoside	Flavones	+	+	+	−
17	10.2	C_27_H_30_O_15_	593.15	−3.6	90	285	Luteolin hexoside deoxyhexoside 1	Flavones	+	+	+	−
18	10.5	C_27_H_30_O_15_	593.15	−3.6	92	447, 285	Luteolin hexoside deoxyhexoside 2	Flavones	+	+	+	−
19	10.6	C_31_H_42_O_18_	701.23	−1.9	96	539, 377, 307, 275	Oleuropein hexoside 1	Phenylethanoids (linked to secoiridoids)	+	+	+	−
20	10.7	C_29_H_36_O_15_	623.20	−3.7	90	461	Isoverbascoside	Caffeoyl phenylethanoid derivatives	+	+	+	−
21	10.8	C_17_H_20_O_7_	335.11	−4.0	93	317, 199, 153, 111	Hydroxyde (carboxymethyl) oleuropein aglycone	Phenylethanoids (linked to secoiridoids)	−	−	+	−
22	11.1	C_31_H_42_O_18_	701.23	−2.4	93	539, 437, 377, 307, 275	Oleuropein hexoside 2	Phenylethanoids (linked to secoiridoids)	+	+	−	−
**23**	**11.2**	C_30_H_34_O_11_	569.20	−2.6	95	**551, 539, 393, 373, 177, 162**	**G(8-O-4)S(8-5)G ^5^ (−C)**	**Lignan derivatives**	−	−	−	+
24	11.3	C_27_H_30_O_14_	557.16	−3.7	90	269	Apigenin 7-*O*-rutinoside	Flavones	+	+	−	−
25	11.4	C_25_H_32_O_13_	539.18	−4.6	85	403, 223	Oleouropein 1	Phenylethanoids (linked to secoiridoids)	−	−	+	−
26	11.5	C_21_H_20_O_11_	447.09	−3.5	91	285	Luteolin 7-*O*-hexoside 1	Flavones	+	+	−	−
27	11.6	C_25_H_32_O_13_	539.18	−3.1	92	403, 377, 307, 275, 223	Oleuropein	Phenylethanoids (linked to secoiridoids)	+	+	+	+
28	11.7	C_25_H_28_O_14_	551.14	−3.6	92	507, 389, 341, 281, 251, 179, 161	Caffeoyl-6′-secologanoside	Hydroxycinnamics (linked to secoiridoids)	−	−	+	−
29	12.0	C_22_H_22_O_11_	461.11	−4.2	90	446, 299, 284	Diosmetin 7-*O*-glucoside	Flavones	+	+	−	−
30	12.3	C_21_H_20_O_11_	447.09	−3.5	92	285	Luteolin 7-*O*-hexoside isomer 2	Flavones	+	+	−	−
31	12.4	C_25_H_32_O_13_	539.18	−2.9	92	377, 307, 275, 223	Oleouropein 2	Phenylethanoids (linked to secoiridoids)	+	+	+	−
32	12.7	C_25_H_32_O_13_	539.18	−3.5	92	403, 377, 307, 275, 223	Oleouropein 3	Phenylethanoids (linked to secoiridoids)	+	+	+	−
33	13.0	C_27_H_36_O_14_	583.20	−2.1	95	537, 403, 223, 179	Lucidumoside C	Phenylethanoids (linked to secoiridoids)	+	+	−	−
34	13.1	C_17_H_20_O_6_	319.12	−3.6	94	183, 181, 153, 111	3,4-DHPEA-EDA	Phenylethanoids (linked to secoiridoids)	−	−	+	−
35	13.2	C_19_H_22_O_8_	377.13	−4.3	91	307, 275	Oleuropein aglycone 1	Phenylethanoids (linked to secoiridoids)	+	−	−	−
36	13.2	C_25_H_28_O_13_	535.15	−4.0	90	491, 389, 345, 265, 163	*p*-Coumaroyl-6′-secologanoside	Hydroxycinnamics (linked to secoiridoids)	−	−	+	+
37	13.5	C_25_H_32_O_12_	523.18	−2.9	94	361, 291, 259, 223	Ligustroside	Phenylethanoids (linked to secoiridoids)	+	+	+	−
38	14.1	C_19_H_22_O_8_	377.13	−4.0	91	307, 275	Oleuropein aglycone 2	Phenylethanoids (linked to secoiridoids)	+	−	−	−
**39**	**14.4**	C_21_H_20_O_6_	367.12	−3.7	95	**352, 337, 336, 322, 307, 177, 162**	**S(8-5)G ^4^ derivative 1 (−H_2_O)**	**Lignan derivatives**	−	−	−	+
**40**	**14.6**	C_20_H_18_O_5_	337.11	−3.9	93	**322, 307, 291, 177, 162**	**S(8-5)G ^4^ derivative 2 (−H_2_O, −CH_2_O)**	**Lignan derivatives**	−	−	−	+
41	15.0	C_28_H_34_O_13_	577.19	4.4	92	531, 415, 398, 285, 273, 239	(+)-1-Acetoxypinoresinol 4′-b-*O*-glucoside	Lignan derivatives	+	−	−	−
42	15.4	C_31_H_28_O_14_	623.14	−2.2	94	323, 299, 285	Diosmetin di-hexoside	Flavones	+	+	−	−
43	15.4	C_42_H_54_O_23_	925.30	0.9	97	539, 377, 307, 275	Jaspolyoside isomer 1	Phenylethanoids (linked to secoiridoids)	+	−	−	−
**44**	**15.8**	C_31_H_36_O_11_	583.22	−3.2	93	**565, 535, 413, 387, 373, 357, 343, 195, 165**	**G(8-O-4)S(8-8)G ^5^**	**Lignan derivatives**	−	−	−	+
**45**	**16.2**	C_31_H_34_O_11_	581.20	−2.9	93	**563, 551, 533, 503, 385, 367, 355, 337, 336, 218, 195, 177, 165**	**G(8-O-4)S(8-5)G ^4^ 1**	**Lignan derivatives**	−	−	−	+
46	16.4	C_41_H_50_O_21_	877.28	0.2	99	715, 701, 539, 377, 307, 275, 149	Oleuropein derivative 1 (oleuropein hexoside + C_10_H_8_O_3_)	Phenylethanoids (linked to secoiridoids)	+	+	−	−
47	16.9	C_42_H_54_O_23_	925.30	0.6	96	539, 377, 307, 275	Jaspolyoside isomer 2	Phenylethanoids (linked to secoiridoids)	+	−	−	−
48	17.0	C_31_H_34_O_11_	581.20	−2.6	94	**551, 367, 355, 337, 218, 195, 165**	**G(8-O-4)S(8-5)G ^4^ 2**	**Lignan derivatives**	−	−	−	+
49	17.0	C_34_H_38_O_15_	685.21	−1.7	92	539, 377, 307, 275	Oleuropein derivative 2 (oleuropein + C_9_H_6_O_2_)	Phenylethanoids (linked to secoiridoids)	+	−	−	−
50	18.3	C_41_H_58_O_20_	869.34	0.6	95	829, 707, 539, 377, 325, 307, 275, 145	Oleuropein derivative 3 (oleuropein hexoside + C_10_H_16_O_2_)	Phenylethanoids (linked to secoiridoids)	+	−	−	−
**51**	**18.7**	**C_19_H_22_O_7_**	**361.13**	**−1.6**	**98**	**329, 291, 225, 193, 181**	**Hydroxytyrosol linked to desoxy elenolic acid**	**Phenylethanoids (linked to secoiridoids)**	−	−	**+**	−
52	19.3	C_35_H_48_O_15_	707.29	−0.5	97	539, 377, 307, 275	Oleuropein derivative 4 (oleuropein + C_10_H_16_O_2_)	Phenylethanoids (linked to secoiridoids)	+	−	−	−
53	26.2	C_43_H_60_O_14_	799.39	0.04	99	539, 377, 307, 277	Oleuropein derivative 6 (oleuropein + C_18_H_28_O)	Phenylethanoids (linked to secoiridoids)	+	−	−	−
54	26.8	C_41_H_62_O_14_	777.41	0.23	98	539, 377, 307, 275	Oleuropein derivative 7 (oleuropein + C_16_H_30_O)	Phenylethanoids (linked to secoiridoids)	+	−	−	−
55	14.2	C_15_H_10_O_6_	285.04	−2.12	98	175, 151	Luteolin	Flavones	−	+	+	−

+ Presence; − absence; bold letter indicates novel compounds. ^1^ By RP-HPLC-IT-MS. ^2^ By RP-HPLC-QTOF-MS. ^3^ [M−H]^−^ ions. ^4^ G is coniferyl alcohol; S, sinapyl alcohol; G′ is coniferyl aldehyde. The first coniferyl alcohol is hydroxylated (+O). ^5^ G is coniferyl alcohol; S, sinapyl alcohol. The first coniferyl alcohol is hydroxylated (+O).

**Table 4:** Non-phenolic compounds characterized in the extracts from olive leaves (OL), olive mill leaves (OML), exhausted olive pomace (EOP), and residual fraction from olive pit cleaning (RFOPC) obtained by ultrasound-assisted extraction.

No	RT ^1^ (Min)	Molecular Formula ^2^	*m*/*z*^2,3^	Error ^3^ (Ppm)	Score ^3^	MS/MS Fragments ^1^	Compound	OL	OML	EOP	RFOPC
**1′**	**0.4**	C_6_H_14_O_6_	181.072	-0.2	99	163, 119	Mannitol	+	+	+	+
2′	0.5	C_6_H_8_O_7_	191.020	−0.7	99	173, 111	Citric acid	+	+	+	+
3′	0.5	C_7_H_12_O_6_	191.057	−2.1	99	Quinic acid	+	+	+	+
4′	0.5	C_6_H_12_O_7_	195.052	−2.9	98	177, 129	Gluconic acid	+	+	−	+
**5′**	**1.6**	**C_17_H_28_O_11_**	**407.158**	**−4.5**	**90**	**389, 375, 357, 313, 151**	**Loganin derivative (+H_2_; +O)**	**+**	**−**	**+**	**−**
6′	3.7	C_12_H_18_O_7_S	305.071	−3.9	94	225, 97	12−Hydroxyjasmonic acid sulfate	+	+	−	−
7′	2.5	C_16_H_22_O_11_	389.110	−2.8	93	345, 302, 209, 165, 139, 121	Oleoside/Secologanoside	+	+	+	+
**8′**	**3.3**	**C_9_H_12_O_4_**	**183.066**	**0.8**	**99**	**139**	**Oleoside/secologanoside derivative (-glucosyl; −CO_2_) or decarboxymethylelenolic acid**	**−**	+	+	+
9′	3.9	C_16_H_22_O_11_	389.111	−4	91	345, 302, 209, 187, 165, 139, 121	Oleoside/Secologanoside	−	−	+	−
**10′**	**4.4**	**C_18_H_28_O_12_**	**435.153**	**−4.1**	**90**	**389, 357, 313, 151**	**Loganin derivative (+H_2_; +O; +CO)**	**−**	**−**	**+**	**−**
11′	4.7	C_17_H_24_O_11_	403.13	−4.3	90	371, 223, 179	Elenolic acid hexoside 1	+	−	−	−
**12′**	**5.3**	**C_12_H_20_O_7_S**	**307.09**	**−4.4**	**92**	**227, 165, 97**	**Dihydrohydroxyjasmonic acid sulfate derivative**	**−**	**−**	**+**	**−**
**13′**	**5.3**	**C_10_H_14_O_4_**	**197.08**	**0.7**	**98**	**153**	**Elenolic acid hexoside derivative (+H_2_; -CO; -Hexose)**	**−**	**−**	**−**	**+**
**14′**	**5.5**	**C_16_H_26_O_10_**	**377.15**	**−4.4**	**90**	**197, 153**	**Elenolic acid hexoside derivative (+H_2_; -CO)**	**+**	**+**	**+**	**+**
**15′**	**6.2**	**C_17_H_26_O_11_**	**405.14**	**−4.7**	**90**	**373, 181**	**Elenolic acid hexoside derivative (+H_2_)**	**−**	**−**	**+**	**−**
16′	6.5	C_17_H_24_O_11_	403.13	−4	92	371, 223, 179	Elenolic acid hexoside 2	+	+	+	−
17′	6.9	C_20_H_34_O_13_	481.19	−3.9	90	371, 151	Unknown (elenolic acid hexoside derivative)	**−**	**−**	**+**	**−**
18′	7.5	C_11_H_14_O_7_	257.07	−1	99	239, 225, 195, 137	Hydroxyelenolic acid	**−**	**−**	+	+
19′	7.8	C_17_H_24_O_11_	403.13	−4.2	92	241, 223	Elenolic acid hexoside 3	+	+	+	−
20′	9.3	C_9_H_16_O_4_	187.1	−1	99	125	Azelaic acid	−	+	−	+
**21′**	**11.6**	**C_18_H_34_O_6_**	**345.23**	**−2.5**	**96**	**201, 171**	**Tetrahydroxyoctadecenoic acid isomer 1**	**−**	**−**	**−**	**+**
22′	**12.1**	**C_18_H_34_O_6_**	**345.23**	**−3.4**	**95**	**327, 309, 201, 171**	**Tetrahydroxyoctadecenoic acid isomer 2**	**−**	**−**	**−**	**+**
23′	**12.4**	**C_18_H_34_O_6_**	**345.23**	**−4.2**	**91**	**327, 309, 201**	**Tetrahydroxyoctadecenoic acid isomer 3**	**−**	**−**	**−**	**+**
**24′**	15.5	C_26_H_38_O_13_	557.23	3.6	91	513, 345, 227, 185	6′-*O*-[(2E)-2,6-Dimethyl-8-hydroxy-2-octenoyloxy]-secologanoside	+	+	+	−
25′	15.8	C_18_H_32_O_5_	327.22	−3.8	93	201, 171	Trihydroxyoctadecadienoic acid	−	−	−	+
26′	**16.4**	**C_18_H_32_O_6_**	**343.21**	**−3.8**	**93**	**325, 307, 245, 201**	**Dihydroxyoctadecenedioic acid**	−	−	−	**+**
27′	16.7	C_18_H_34_O_5_	329.23	−2.4	95	201	Trihydroxyoctadecenoic acid	−	−	+	+
28′	17.7	C_18_H_34_O_6_	345.23	−4.2	91	327, 309, 265, 247	Dihydroxyoctadecanedioic acid	−	−	−	+
29′	18.1	C_18_H_36_O_5_	331.25	−4	92	312.7	Trihydroxyoctadecanoic acid	−	+	+	+
**30′**	18.9	C_16_H_32_O_4_	287.22	−3.3	95	269, 210	Dihydroxyhexadecanoic acid	+	+	+	+
**31′**	21.7	C_30_H_48_O_5_	487.34	−3.8	90	467	Hydroxylated derivative of maslinic acid	−	−	−	+
**32′**	**21.2**	**C_20_H_36_O_6_**	**371.25**	**−3.4**	**93**	**329, 311, 201**	**Trihydroxyoctadecenoic acid + C_2_H_2_O (acetyl)**	**−**	**−**	**+**	**+**
33′	**24.7**	**C_36_H_64_O_9_**	**639.45**	**−2.9**	**93**	**329, 327, 201**	**Dimer formed by trihydroxyoctadecadienoic acid and trihydroxyoctadecenoic acid**	**−**	**−**	**+**	**+**
**34′**	**24.9**	**C_36_H_66_O_9_**	**641.47**	**−2.3**	**93**	**329, 201**	**Di-trihydroxyoctadecenoic acid**	**−**	**−**	**+**	**+**
35′	25.1	C_30_H_48_O_4_	471.35	−3.4	94	453, 407	Pomolic acid	+	+	−	−
36′	**25.3**	**C_34_H_64_O_8_**	**599.45**	**−2.7**	**94**	**329, 287, 201**	**Dimer formed by trihydroxyoctadecenoic acid and dihydroxyhexadecanoic acid**	**−**	**−**	**+**	**+**
37′	25.7	C_30_H_48_O_4_	471.35	−3.3	93	423, 405	Maslinic acid	+	+	+	+
38′	26	C_18_H_36_O_3_	299.26	−2.9	95	281, 253	Hydroxyoctadecanoic acid	−	−	−	+
39′	26.9	C_30_H_48_O_3_	455.35	−3.4	93	407, 395	Oleanolic acid	+	+	+	+

+ Presence; − absence; Bold letter indicates novel compounds. ^1^ By RP-HPLC-IT-MS. ^2^ By RP-HPLC-QTOF-MS. ^3^ [M−H]^−^ ions.

**Table 5:** Amount of protein solubilized (mg/100 g of byproduct, dry basis) and recovery (%, dry basis) obtained after the integration of antioxidants and alkaline extraction under mild (NaOH 0.4 M, 80 °C, 4 h) and strong alkaline conditions (NaOH 0.7 M, 100 °C, 4 h).

Byproduct ^1^	Mild	Strong	Mild	Strong
	Solubilized Protein (g/L)	Solubilized Protein (g/L)	Recovery (%)	Recovery (%)
OML ^2^	3.9 ± 0.1 ^b^	5.1 ± 0.5 ^a^	48.7 ± 1.5 ^c^	63.1 ± 5.7 ^b^
OL ^2^	ND	5.2 ± 0.4	ND	55.5 ± 4.3 ^b^
EOP	6.0 ± 0.3 ^a^	5.4 ± 0.4 ^a^	63.9 ± 2.3 ^b^	58.2 ± 3.3 ^b^
RFOPC	3.7 ± 0.3 ^b^	4.5 ± 0.2 ^a^	81.3 ± 5.8 ^a^	100.1 ± 3.0 ^a^

Different lowercase letters within a row indicate significant differences between the samples (*p* < 0.05).^1^ EOP, extracted olive pomace; OL, olive leaves; OML, olive mill leaves; RFOP, residual fraction from olive pomace. ^2^ Data from [14,21]; ND, not determined.

## Data Availability

The data presented in this study are available on request from the corresponding author. The data are not publicly available due to privacy concerns.

## Supplementary Materials

The following are available online at https://www.mdpi.com/2304-8158/10/1/111/s1, Table S1: Pearson correlation values for the solubilization of phenolic compounds, Figure S1: UV-Vis spectra and their first derivative of alkaline extracts from the extracted olive pomace (EOP) (a and c, respectively) and the residual fraction from olive pit cleaning (RFOPC) (b and d, respectively).

## Abbreviations

EOP	extracted (or exhausted) olive pomace
FRAP	ferric ion reducing antioxidant power
HPLC	high performance liquid chromatography
IT	ion trap
MS	mass spectrometry
OML	olive mill leaves
OL	olive leaves
ORAC	oxygen radical absorbance capacity
QTOF	quadrupole-time-of-flight
RFOPC	residual fraction from olive pit cleaning (or residual pulp)
RP	reversed phase
TEAC	Trolox equivalent antioxidant capacity
TPC	total phenol content
UAE	ultrasound-assisted extraction
