# Supplementary material for: Olive Pomace-Derived Biomasses Fractionation through a Two-Step Extraction Based on the Use of Ultrasounds: Chemical Characteristics
Source: Foods. 2021 Jan 7;10(1):111. doi: 10.3390/foods10010111 (PMC7825784; doi:10.3390/foods10010111)
Supplement: Supplementary file 1 [file foods-10-00111-s001.pdf]

Type of the Paper (Article)

Fractionation of olive pomace-derived biomasses through a two-step extraction based on the use of ultrasounds: Chemical characteristics

María del Mar Contreras [1,2,*], Irene Gómez-Cruz [1,2], Inmaculada Romero[1,2] and Eulogio Castro [1,2]

[1] Department of Chemical, Environmental and Materials Engineering and [2] Center for Advanced Studies in Earth Sciences, Energy and Environment (CEACTEMA), University of Jaén, Campus Las Lagunillas, 23071 Jaén, Spain; mcgamez@ujaen.es (M.d.M.C.); igcruz@ujaen.es (I.G.-C.); iromero@ujaen.es (I.R.); ecastro@ujaen.es (E.C.)

* Correspondence: mcgamez@ujaen.es (mar.contreras.gamez@gmail.com) (M.d.M.C.)

Received: date; Accepted: date; Published: date

Supplementary material

Table S1. Pearson correlation values for the solubilization of phenolic compounds.

Component	Ext[1]	Yield [1]	TPC [1]	TEAC [1]	FRAP [1]	ORAC [1]
Extractives	1					
Yield	0.992**	1				
TPC	0.992**	0.983*	1			
TEAC	0.958*	0.954*	0.914	1		
FRAP	0.997*	0.991	0.999*	0.939	1	
ORAC	0.991	0.982	1.000**	0.918	0.999*	1

[1] Ext, extractives content (%); yield, total extraction yield (%); FRAP, ferric reducing antioxidant power assay (mmol Trolox equivalents/100 g); TEAC, trolox equivalent antioxidant capacity (mmol Trolox equivalents/100 g); TPC, total phenolic content (g gallic acid equivalents/100 g); ORAC, oxygen radical absorbance capacity (mmol Trolox equivalents/100 g). **P-value<0.01;*0.01<P-value<0.05.

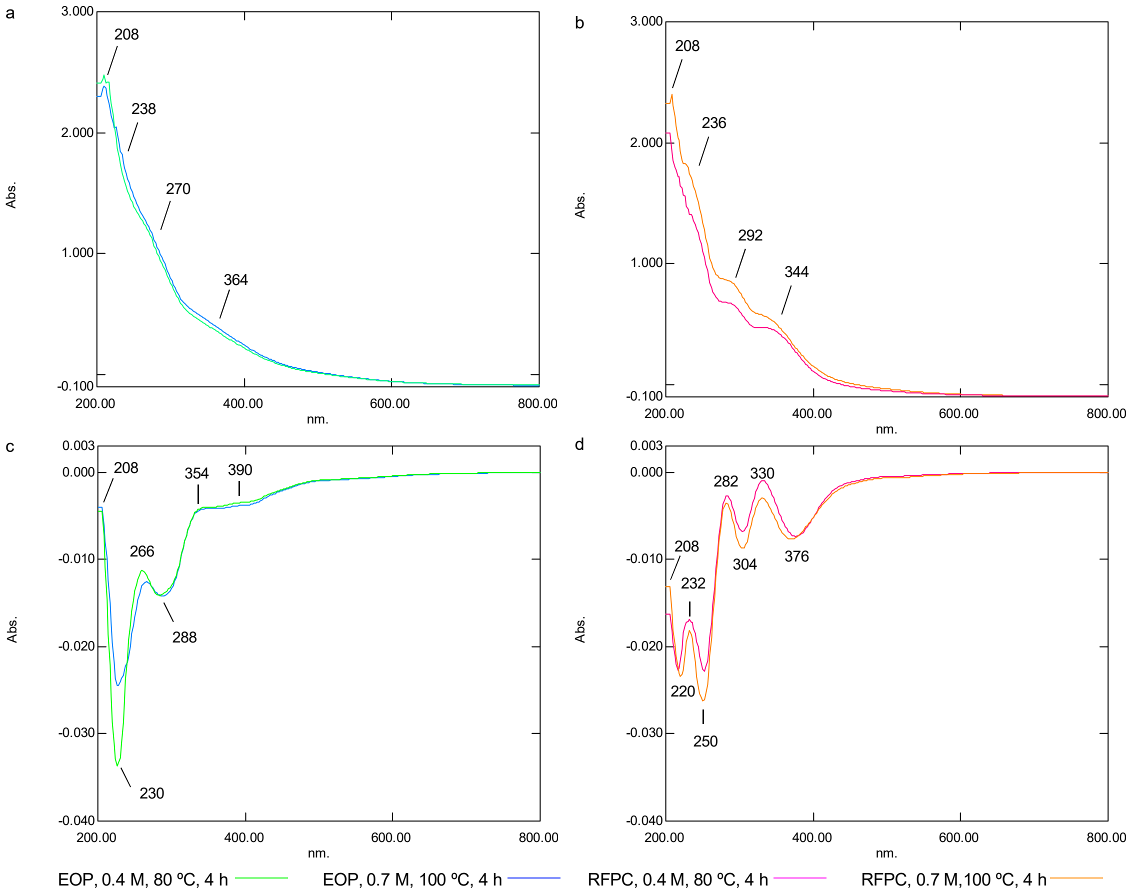

Figure S1. UV-VIS spectra and their first derivative of alkaline extracts from the extracted olive pomace (EOP) (a and c, respectively) and the residual fraction from olive pit cleaning (RFOPC) (b and d, respectively).
